# Fiery Connections: Macrophage-Mediated Inflammation, the Journey from Obesity to Type 2 Diabetes Mellitus and Diabetic Kidney Disease

**DOI:** 10.3390/biomedicines12102209

**Published:** 2024-09-27

**Authors:** Satyesh K. Sinha, Maria Beatriz Carpio, Susanne B. Nicholas

**Affiliations:** 1 Department of Medicine, David Geffen School of Medicine, University of California, Los Angeles, CA 90095, USA; mariablcarpio@gmail.com; 2 Department of Internal Medicine, Charles R. Drew University of Medicine and Science, Los Angeles, CA 90059, USA

**Keywords:** macrophage, monocyte, inflammation, obesity, diabetes, diabetic kidney disease

## Abstract

The high prevalence of diabetes mellitus (DM) poses a significant public health challenge, with diabetic kidney disease (DKD) as one of its most serious consequences. It has become increasingly clear that type 2 DM (T2D) and the complications of DKD are not purely metabolic disorders. This review outlines emerging evidence related to the step-by-step contribution of macrophages to the development and progression of DKD in individuals who specifically develop T2D as a result of obesity. The macrophage is a prominent inflammatory cell that contributes to obesity, where adipocyte hypertrophy leads to macrophage recruitment and eventually to the expansion of adipose tissue. The recruited macrophages secrete proinflammatory cytokines, which cause systemic inflammation, glucose dysregulation, and insulin sensitivity, ultimately contributing to the development of T2D. Under such pathological changes, the kidney is susceptible to elevated glucose and thereby activates signaling pathways that ultimately drive monocyte recruitment. In particular, the early recruitment of proinflammatory macrophages in the diabetic kidney produces inflammatory cytokines/chemokines that contribute to inflammation and tissue damage associated with DKD pathology. Macrophage activation and recruitment are crucial inciting factors that also persist as DKD progresses. Thus, targeting macrophage activation and function could be a promising therapeutic approach, potentially offering significant benefits for managing DKD at all stages of progression.

## 1. Introduction

The prevalence of diabetes mellitus (DM) poses a significant public health challenge and is projected to reach critical levels in the coming decades [1,2]. In 2022, it was estimated that 537 million adults (20–79 years old) were living with DM. This number is projected to increase to 643 million by 2030 and 781 million by 2045, representing a significant rise across all regions of the world [1,2]. Diabetic kidney disease (DKD) is a complication of both type 1 and type 2 DM (T1D and T2D, respectively) that is already widely recognized as a global medical catastrophe and is the leading cause of end-stage kidney disease (ESKD) [3]. Over the past decade, noteworthy advancements in knowledge of the mechanisms involved in the development of T2D, which accounts for over 90% of all cases of DM [4,5], and its complications have reshaped our understanding of the pathogenesis of the disease. It has become increasingly clear that T2D and its associated conditions are not purely metabolic disorders; rather, numerous inflammatory molecules significantly contribute to the initiation and progression of DKD [6,7,8]. Among the pivotal cells involved, macrophages stand out as key players in the pathogenesis of both T2D and DKD. In this review, we explore current knowledge of the process of macrophage-driven inflammation from its earliest manifestation in T2D stemming from obesity, through its contribution to DKD and DKD progression to ESKD, as a pathway to “better understand the role of the macrophage in the development of disease”.

## 2. Macrophage and Adiposity Are Major Risk Factors for T2D

**Figure 1:**
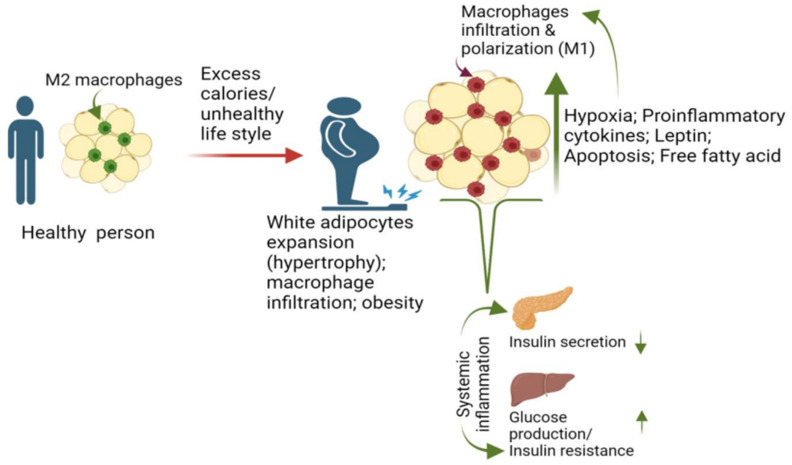
**Macrophage and adiposity are major risk factors for T2D**. Diet and lifestyle influence adipose tissue hypertrophy, leading to the recruitment and proliferation of macrophages, particularly through elevated levels of free fatty acids, hypoxia, leptin, and adipocyte apoptosis. The proinflammatory macrophages (M1) then contribute to systemic inflammation (proinflammatory cytokines), glucose regulation issues, and insulin resistance. T2D—type 2 diabetes mellitus.

Impaired glucose tolerance and T2D are believed to result from prolonged exposure to lifestyle or environmental triggers that disrupt normal physiological responses, leading to maladaptation and disease [9,10,11]. For example, in a cohort of 84,941 female nurses followed from 1980 to 1996, a total of 91 percent of the cases of T2D were attributed to poor diet and sedentary lifestyle [12]. The effects of such a lifestyle on adipose tissues begin with the recruitment and proliferation of macrophages from circulation [13]. Furthermore, the discovery of leptin, an adipose-specific cytokine-like molecule [14] highlights the active role of adipose tissue in metabolic processes in response to environmental factors [15]. Leptin engages several important pathways, like mitogen-activated protein kinases (MAPK), phosphatidylinositol 3′ -kinase (PI3K)/AKT, janus kinase/signal transducer and activator of transcription (JAK/STAT), transforming growth factor beta/AMP-activated protein kinase (TGF-β/AMPK), wingless-related integration site/β-catenin, glucagon-like peptide 1 (GLP-1) and melanocortin [16], in this process. By using mice lacking leptin, deficient in leptin receptors, and those with diet-induced obesity, researchers illustrated heightened levels of macrophage markers in white adipose tissue (WAT) compared to their respective control groups [17,18]. It was also determined that the macrophages were mainly derived from bone marrow [18]. Similarly, the quantity of macrophages in adipose tissue rises during obesity and contributes to the activation of inflammatory pathways within the adipose tissues of obese individuals [18]. These initial studies were further validated by multiple research endeavors confirming the presence of macrophage infiltration during the development of obesity [13,19,20,21]. This process paves the way for elucidating the role of macrophages in an inflammation-related pathway in adipose tissue (Figure 1).

### 2.1. Macrophage Recruitment and Proliferation in Adipose Promotes T2D

In the case of obesity, continuous overnutrition leads to lipid accumulation and hypertrophy of WAT [22,23], one of the major factors responsible for macrophage recruitment. The enlarged adipocytes secrete numerous chemokines, attracting immune cells (e.g., neutrophils, eosinophils, dendritic cells, mast cells, natural killer T and B cells) and, notably, macrophages [24], which trigger chronic low-grade inflammation, insulin resistance, and the substantial release of free fatty acids (FFAs) into the circulation. This cascade ultimately results in metabolic dysfunction and further macrophage recruitment [25,26,27,28,29]. Thus, adipose tissue can be perceived as both an immune and a secretory organ, with obesity representing an inflammatory immune disorder [29]. Adipocyte hypertrophy in obesity represents a multifaceted process characterized not only by the enlargement of individual cells but also by the restructuring of adipose tissue [30]. Additionally, hypertrophy imposes mechanical stress, as well as oxygen shortage, resulting in adipocyte death [31]. Experiments with parabiotic mice, which are surgically paired mice sharing a common circulatory system, confirmed that adipocyte apoptosis initiates a distinct degradation process and locally induces a metabolically active proinflammatory macrophage (M1) phenotype [32]. These M1 macrophages secrete proinflammatory mediators, such as tumor necrosis factor (TNF)-α and interleukin (IL)-1β [32,33,34], that, in turn, stimulate inflammatory pathways, such as the c-Jun N-terminal kinase (JNK) or inhibitor of nuclear factor kappa-B kinase subunit (IKK)β pathways in adipocytes [35,36]. Hypertrophy also leads to various outcomes, such as heightened leptin secretion, increased free fatty acid flux, vascularization, hypoxia, and adipocyte cell death [13]. These consequences, due to adipose tissue expansion, are potential factors initiating the recruitment of macrophages [13]. Besides the recruitment of circulating monocytes, another factor contributing to the increased accumulation of adipose tissue macrophages (ATMs) is the local proliferation of macrophages in adipose tissue [26,37,38,39]. As mentioned above, the recruited macrophages secrete numerous proinflammatory cytokines, establishing a systemic inflammatory milieu, and subsequently impacting glucose regulation and insulin resistance [40] (Figure 1).

#### 2.1.1. Elevated Leptin Levels Contribute to the Accumulation and Activation of Macrophages in Adipose

Leptin, the primary classical adipokine, was initially recognized as a satiety signal responsible for regulating body weight by decreasing food intake and boosting energy expenditure [41,42]. Mice lacking leptin or its receptor exhibit severe obesity due to overeating [43]. Macrophages derived from these mice exhibited reduced phagocytosis and inflammatory cytokine production. Conversely, administration of exogenous leptin resulted in the upregulation of both functions [44,45,46]. Similarly, leptin treatment of human macrophages induces the expression of M2 markers while also increasing the expression of M1 markers [47]. Furthermore, leptin triggers the production of proinflammatory cytokines by activating the JAK2-STAT3 pathway in murine macrophages [46,48].

These findings indicate that leptin may influence the phenotype of ATMs. Additionally, leptin promotes the expression of vascular cell adhesion molecules, facilitating macrophage infiltration into adipose tissue [49]. However, 12 weeks of leptin treatment in leptin-deficient low-density lipoprotein receptor knockout female mice reduced macrophage infiltration in WAT and lowered mRNA levels of pro-inflammatory IL-6 and macrophage chemotactic protein (MCP-1) in different WAT depots and brown adipose tissue, along with decreased IL-6 and MCP-1 plasma levels [50]. Using two mouse models of partial leptin deficiency, OBHZ, which are whole-body leptin heterozygous mice, and LepHZ, which are adipocyte-specific congenital heterozygous leptin knockout mice, researchers found that when exposed to a high-fat diet (HFD), the inability to increase leptin levels due to partial leptin deficiency helped protect against diet-induced obesity and metabolic problems [42,51,52]. Therefore, in a setting where obesity is likely, keeping leptin levels lower seems to partially control both obesity and T2D [42,52]. Higher serum leptin levels were found in a study of patients with T2D [53] while a lower adiponectin/leptin ratio correlated with higher body mass index (BMI), body fat, waist-to-height ratio, and plasma resistin, indicating potential early insulin resistance in obese adults [54]. Likewise, in obese women, serum leptin levels were elevated, which decreased with weight loss [55]. Moreover, leptin stimulates macrophage proliferation in a dose-dependent manner, suggesting that high levels of leptin associated with obesity can enhance the proliferation and activation of ATMs [51,56].

#### 2.1.2. Increased Free Fatty Acid (FFA) Flux Promotes Macrophage Recruitment in Adipose

Hypertrophic adipocytes face various harmful factors such as lipotoxicity, which impairs insulin function and leads to increased release of FFA [57,58]. This contributes to ectopic fat accumulation in organs, like the liver, skeletal muscle, heart, and pancreas, leading to systemic insulin resistance [59]. In a study using male C57BL/6J mice, it was reported that an increase in ATMs coincides with elevated levels of circulating FFA and adipose tissue lipolysis. Manipulations that reduce lipolysis in these mice also decrease the recruitment of ATMs, suggesting that FFA drives ATM accumulation [60,61]. Plasma FFA is elevated in most obese individuals, particularly after consuming a high-fat meal [62]. Furthermore, lipoxygenases (LO) are enzymes involved in fatty acid inflammation and insulin resistance. Knockout mice lacking 12/15LO showed reduced macrophage recruitment and IR caused by HFD, indicating the role of 12/15LO in macrophage recruitment and IR development [61,63]. Conditioned media from human adipocytes modulate the expression profile of macrophages by increasing angiotensin-converting enzyme expression via AMPK. This prevents pro-inflammatory cytokine production by macrophages [64].

Han et al. [65] demonstrated in vitro that high glucose (HG) concentrations and saturated FFA regulate the expression of MCP-1 via a mechanism involving the generation of reactive oxygen species (ROS) and the activation of inflammatory pathways, such as nuclear factor κB (NF-κB) and peroxisome proliferator-activated receptor gamma. This regulatory process was counteracted by polyunsaturated FFA, which inhibits these monocyte recruitment pathways [65]. Specific saturated FFA, unlike excessive glucose, induces chemotactic factor expression via a toll-like receptor-4 (TLR-4)-dependent pathway. TLR-4 binds to FFA, like palmitate and stearate indirectly, facilitated by an endogenous ligand, such as Fetuin-A [66]. TLR-4 mediates NF-κB activation leading to the production of pro-inflammatory cytokines and prostaglandins through increased expression of cyclooxygenase-2 [67,68]. These pieces of evidence suggest that increased FFA flux promotes macrophage recruitment and inflammation in adipose tissue. As such, targeting TLR-4 signaling inhibition has emerged as a promising therapeutic strategy for managing IR induced by obesity [61].

#### 2.1.3. Hypoxia Promotes Macrophage Infiltration in Adipose

During the progression of obesity, the rapid expansion of adipose tissue and enlargement of adipocytes result in a decrease in oxygen levels within the tissue, known as adipose tissue hypoxia [46,69]. This leads to the activation of hypoxia-inducible factor 1-alpha (HIF1-α), which in turn stimulates the production of extracellular matrix (ECM) proteins [69,70]. Chronic hypoxia is also implicated in adipocyte dysfunction [70,71], affecting lipid metabolism and lipoprotein clearance [72]. Studies suggest that hypoxia increases lipolysis through various mechanisms, including reduced lipolytic signaling efficiency [73] and activation of protein kinase A [74]. Adipocytes, both murine and human, undergo significant functional changes in culture in response to hypoxia, altering the expression of genes related to the upregulation of adipokine genes, such as leptin, IL-6, vascular endothelial growth factor, and matrix metalloproteinase-2, and downregulation of adiponectin [75]. Adiponectin is recognized for its anti-inflammatory and anti-diabetic properties, and its levels decrease in obesity, metabolic syndrome, and T2D [76,77]. It suppresses pro-inflammatory cytokines, like TNF-α and IL-6 [78], and promotes the anti-inflammatory M2 polarization of macrophages in both humans and mice [79]. Research has shown that inflammatory factors from human M1 macrophages can downregulate the expression of the adiponectin gene in human adipocytes [80]. Interestingly, a recent study in T2D patients found that adiponectin was positively associated with FFA under certain conditions, suggesting a potential link between adiponectin and FFA-related inflammation [81]. Furthermore, hypoxia, through the induction of HIF1-α, promotes the expression of various proinflammatory genes, exacerbating low-grade inflammation and contributing to the worsening of adipose tissue fibrosis [82]. Furthermore, the enlargement of adipocytes and the hypoxic conditions in adipose tissue are closely linked to an increased infiltration of macrophages, which in turn promotes further accumulation of ECM proteins locally [30,83]. Within fibrotic adipose tissue, there is a significant presence of M1 macrophages [46]. These macrophages, apart from producing proinflammatory cytokines, secrete cytokines, such as TGF-β1 and platelet-derived growth factor, directly stimulating fibroblasts and enhancing ECM accumulation [84]. This sets off a harmful cycle in which macrophages further drive fibrogenesis by releasing chemokines that attract more fibroblasts and proinflammatory cells [85]. Additionally, macrophage-inducible C-type lectin, induced in macrophages by TLR-4 activation, plays a role in regulating ECM production and degradation as well as fibroblast proliferation [86,87].

In addition to infiltration, the other sources of adipose tissue macrophages are tissue-resident macrophages [26]. In mice, most tissue-resident macrophages originate from yolk sac primitive precursors and help regulate tissue remodeling and maintain homeostasis [88]. However, a recent fate-mapping study revealed that adipose tissue-resident macrophages are derived from definitive embryonic hematopoietic precursors [89]. In response to HFD, a subset of ATMs were rapidly increased in adipose tissue and replenished by bone marrow-derived monocytes, implying that recruited monocytes are the major cells contributing to increased ATMs in obesity [89]. Thus, the signaling pathways involved in hypoxia may provide a new target for the treatment of obesity-associated disorders, including T2D.

## 3. Contribution of Adipose Macrophage Activation to the Development of T2D

It has become evident that adipose tissue is a highly active tissue, homing inflammatory macrophages that synthesize and release inflammatory cytokines, such as TNF-α, IL-1, and IL-6 [11,26]. These proinflammatory cytokines play a role in various metabolic pathways relevant to IR, including insulin regulation, ROS, lipoprotein lipase activity, and adipocyte function [11,26]. The inflammatory cytokines also induce β-cell dysfunction through chronic, low-grade inflammation and impair insulin signaling in peripheral tissues in T2D [90]. Similarly, in T1D, macrophages infiltrate the pancreatic islets and secrete cytokines, such as TNF-α and IL-1β, contributing to the destruction of β-cells [91]. Thus, the macrophage-mediated inflammatory response is central to the progression of both forms of DM, exacerbating insulin resistance and β-cell loss [90,91]. Macrophage infiltration into the kidneys begins early in T2D and coincides with the onset of injury [92]. In DM, the kidney is particularly susceptible to pathological changes caused by elevated glucose levels, advanced glycation end products, angiotensin II, and oxidative stress [93]. These factors specifically impact kidney tissue by activating signaling pathways that lead to the production of chemokines and cytokines, such as MCP-1 and CSF-1, which drive monocyte recruitment and inflammation [94]. Hyperglycemia further intensifies this process by increasing the expression of cell adhesion molecules (ICAM-1/VCAM-1), facilitating monocyte adhesion and transendothelial migration into the kidney [94]. The persistent M1 macrophage infiltration and the associated inflammation result in decreased renal function [95].

**Figure 2:**
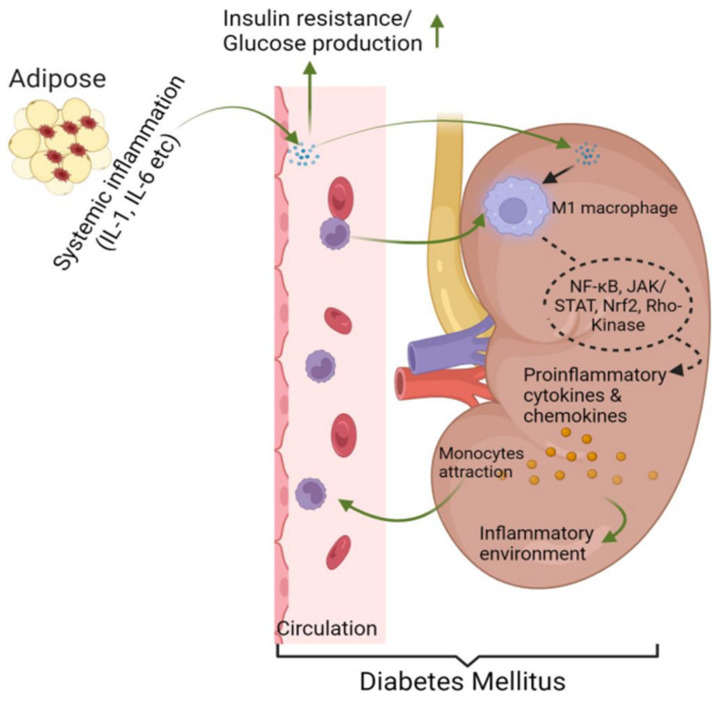
**Contribution of local and recruited macrophage activation in the development of T2D and early DKD.** The recruitment of macrophages to adipose tissue leads to the secretion of proinflammatory cytokines, which trigger systemic inflammation, and increase glucose production and insulin resistance, ultimately contributing to the development of T2D. These inflammatory cytokines in the kidney promote infiltrated macrophages toward an inflammatory phenotype early in T2D, coinciding with initial injury and further reducing renal function by releasing cytokines/chemokines. Pathways, like NF-κB, JAK/STAT, Nrf2, and Rho-kinase, are involved in this process. T2D—type 2 diabetes mellitus, NF-κB—Nuclear factor kappa B, JAK/STAT—Janus kinase/signal transducer and activator of transcription, Nrf2—Nuclear factor erythroid 2-related factor 2.

These macrophages release several inflammatory molecules, including cytokines/chemokines, metalloproteinases, and growth factors, which can worsen kidney damage in T2D [95,96]. Several signaling pathways, like NF-κB, JAK/STAT, nuclear factor erythroid 2-related factor-2, and Rho-kinase, are involved in producing these substances [7]. In diabetic mice, deleting a macrophage scavenger receptor led to protection against albuminuria, expansion of the mesangial matrix, excessive production of TGF-β, and the elevated expression of pro-inflammatory factors [96,97]. Therefore, macrophage activation and inflammation are key factors in the development of T2D, with substantial evidence supporting the presence of a macrophage-mediated inflammatory component [9,11], insulin sensitivity, and an increase in the risk of progression to T2D in high-risk subjects [98] (Figure 2).

Recent advances suggest that prolonged activation of the innate immune system, leading to chronic inflammation, plays a critical role in the development of T2D [9]. Patients with T2D have shown higher levels of circulating inflammatory molecules than healthy controls [10,99,100]. Studies on nondiabetic individuals, those with impaired glucose tolerance, and patients with T2D demonstrate a positive correlation between inflammatory markers and IR [99,101,102,103]. Prospective studies, like the Atherosclerosis Risk in Communities study, have shown that inflammatory markers can predict the onset of T2D [104]. A significant number of subsequent studies have confirmed these findings [105,106,107]. Furthermore, elevated inflammatory markers are strongly associated with T2D risk [108], and anti-inflammatory agents have shown promise in reducing this risk [109,110]. For instance, drugs, like thiazolidinediones, which inhibit macrophage activation and cytokine production, also improve insulin sensitivity and glycemic control [98,109,110]. These findings indicate that macrophages are at the crossroads of the pathogenesis of T2D. Understanding their role in inflammation and polarization is crucial for identifying new therapeutic targets for treating T2D.

## 4. Role of Macrophages in the Progression of DKD

**Figure 3:**
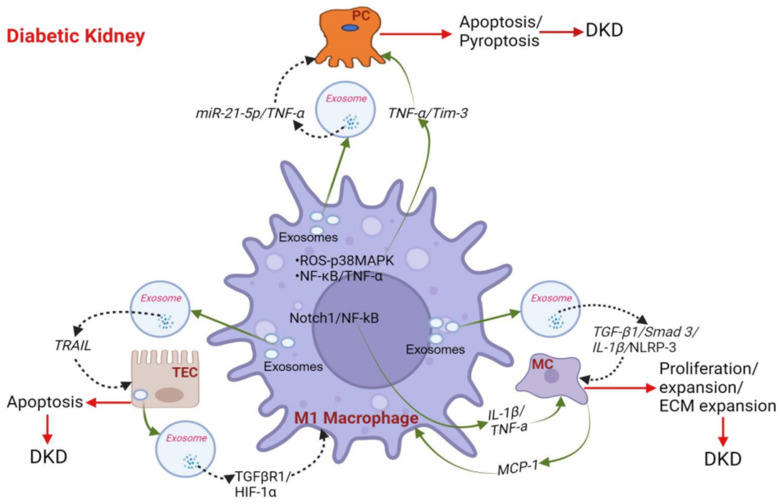
**Role of macrophages in the progression of DKD.** The interaction between macrophages and kidney cells influences the progression of macrophage-mediated inflammation in DKD. Exosomes, nano-sized extracellular vesicles recently identified as cell communication mediators, facilitate intercellular communication. They potentially contribute to podocyte injury, mesangial cell expansion, renal inflammation, and apoptosis. DKD—diabetic kidney disease, TNF-α—tumor necrosis factor α, miR-21-5p—MicroRNA-21-5p, Tim-3—T cell immunoglobulin domain and mucin domain-3, ROS—reactive oxygen species, p38 MAPK—p38 mitogen-activated protein kinases, NF-ΚB—nuclear factor kappa-light-chain-enhancer of activated B, Neurogenic locus notch homolog protein 1, TGF-β1—transforming growth factor beta, SMAD-3—SMAD family member-3, IL-1β—interleukin- beta, NLRP-3—NLR family pyrin domain containing-3, MCP-1—Monocyte chemoattractant protein-1, HIF-1α—hypoxia-inducible factor 1-alpha, PC—podocyte, MC—mesangial cell, TEC—tubular epithelial cell.

In a recent study, the glomerulus tissues of DKD patients exhibited significant alterations in several types of immune cells [111]. Upregulated immune cells included memory B cells, gamma delta T cells, activated natural killer cells, M1 macrophages, M2 macrophages, resting dendritic cells, and resting mast cells. Conversely, downregulated immune cells comprised naïve B cells, resting NK cells, activated mast cells, and neutrophils [111]. However, macrophages are the major cell type contributing to the progression of DKD [111,112]. Cross-talk between macrophages and kidney cells modulates the progression of macrophage-mediated inflammation in DKD [113] (Figure 3).

### 4.1. Macrophages Promote Podocyte Injury and Apoptosis in DKD

Podocytes are highly specialized cells within the glomerulus that play a crucial role in maintaining the integrity of the glomerular filtration barrier. Injury to podocytes represents a central event in the development of DKD [114]. Importantly, macrophages contribute to podocyte injury and apoptosis in DKD, leading to proteinuria [115]. In DKD, HG triggers the release of TNF-α from macrophages through the ROS-p38 MAPK pathway, promoting podocyte apoptosis [116]. Therefore, the loss of podocytes is a key indicator of DKD severity due to their limited regenerative capacity [117,118]. Yang et al. demonstrated that activating the NF-κB/TNF-α signaling pathway in macrophages via the T cell immunoglobulin domain and mucin domain-3 (Tim-3) exacerbates podocyte injury in streptozotocin (STZ)-induced diabetic mice [119]. Also, depleting macrophages in STZ-induced diabetic CD11b-DTR mice (transgenic mice with a diphtheria toxin inducible system that transiently depletes macrophages) significantly reduced albuminuria, kidney macrophage recruitment, and glomerular histological changes, while preserving nephrin and podocin expression [120]. Furthermore, classically activated M1 macrophages increase podocyte permeability, thus affecting their integrity [120]. Another study suggests that sirtuin-6 in macrophages protects podocytes against injury within a simulated diabetic kidney environment, possibly by promoting M2 macrophage activation [121].

Exosomes, newly recognized as mediators of cell communication, are a group of nano-sized extracellular vesicles that facilitate intercellular communication by transferring molecular cargo between cells [122]. They have emerged as important players in the microenvironment of kidney tissue and may contribute to podocyte injury. In vitro studies suggest that macrophage-derived exosomes containing MicroRNA-21-5p enhance podocyte injury through the TNF-α induced protein 3 (A20)-mediated pyroptosis pathway [123]. In addition, induced M2 macrophages may mitigate high glucose-induced podocyte injury by inhibiting dual specificity protein phosphatase 1 expression and activating autophagy through exosomal microRNA-25-3p secretion [123]. Furthermore, exosomes secreted by M2 macrophages alleviate lipopolysaccharide-induced podocyte apoptosis by modulating the microRNA-93-5p/TLR-4 axis [124].

### 4.2. Macrophages Activate Glomerular Mesangial Cells (MCs) in DKD

In DKD, glomerular MCs contribute to kidney damage by overproducing ECM, leading to mesangial expansion and glomerulosclerosis, which subsequently impair kidney function [125]. Macrophage–MC communication in DKD involves a complex network of cytokines, growth factors, ROS, and exosomes contributing to the pathogenesis of the disease.

In one study, HG increased macrophage exosome secretion, which triggered activation, proliferation, and the release of extracellular matrix and inflammatory cytokines by MCs [126]. Furthermore, exosomes from high glucose-treated macrophages with TGF-β knockdown reduced ECM and inflammatory factors in MCs, suggesting TGF-β mRNA in exosomes mediates macrophage–MC communication via the TGF-β/SMAD family member-3 (SMAD-3) pathway [126]. TGF-β1 plays a crucial role in the dynamics of ECM proteins [127,128,129]. Its potent fibrotic effect is mediated intracellularly through SMAD-3 [129,130,131,132]. TGF-β1 activates SMAD-3 by phosphorylation, regulating the transcription of target genes, including those that control ECM proteins [130,133,134] (Figure 3). Additionally, Liu et al. [135] demonstrated that MCs internalized exosomes from HG-treated macrophages, which promoted the activation of the NLR family pyrin domain containing-3 (NLRP-3) inflammasome and an autophagy deficiency, leading to DKD in mice. Moreover, mice injected with exosomes derived from HG-stimulated macrophages showed renal dysfunction and mesangial ECM expansion [135]. In DKD, the interaction between the neurogenic locus notch homolog protein 1 (Notch-1) and NF-κB signaling pathways causes macrophage polarization, resulting in increased cytokine secretion (IL-1β, TNF-α, etc.), which in turn increases the proliferation of MCs, matrix secretion, and glomerulosclerosis. Macrophage depletion and Notch pathway inhibition treatment alleviate these kidney cell pathologies in mice [136]. However, in DKD, communication between macrophages and MCs is bidirectional. For example, increased expression of MCP-1 has been reported in HG-treated MCs [137]. MCP-1 is a potent chemotactic factor that stimulates the recruitment and activation of monocytes and macrophages, and in the kidney, several cell types produce MCP-I, including MCs and podocytes [138]. Yang et al. [139] demonstrated that HG induces rat MC proliferation and MCP-1 expression via ROS-mediated activation of the NF-kB pathway, which is inhibited by eleutheroside, an NF-κB inhibitor. Moreover, Chen et al. [140] reported that increased microRNA-192 expression in rat MCs cultured in HG conditions led to upregulation of MCP-1 expression by inhibiting the expression of zinc finger e-box binding homeobox 1. The importance of MCP-1/C-C chemokine receptor type 2 (CCR-2, the MCP-1 receptor) in attracting macrophages and driving inflammatory responses has been widely acknowledged [141]. Blocking the MCP-1/CCR-2 pathway in diabetic mice using propagermanium, a CCR-2 antagonist, resulted in reduced macrophage infiltration compared to control mice [142]. Furthermore, Ishibashi et al. [143] suggested that GLP-1 downregulated the expression of MCP-1 produced by advanced glycation end products treated with MCs and exerted anti-inflammatory effects. Collectively, these findings support the hypothesis that MCs in an HG environment can recruit macrophages via MCP-1/CCR-2 to enhance inflammatory responses [144].

### 4.3. Cross-Talk between Macrophage and Tubular Epithelial Cells (TECs) in DKD

Communication between tubular epithelial cells (TECs) and macrophages can establish a negative feedback loop through exosomes, resulting in renal inflammation and apoptosis in DKD [145,146,147]. These exosomes are critical in mediating the interaction between TECs and macrophages that regulate macrophage function [148,149]. Jiang et al. demonstrated that TECs from mouse models of T2D increased the release of exosomes when treated with lysophosphatidylcholine (LPC) [145]. Notably, TEC-derived exosomes activated an inflammatory phenotype in macrophages and triggered the release of macrophage-derived exosomes. Moreover, these macrophage-derived exosomes induced apoptosis in LPC-injured TECs [145]. Importantly, researchers found that leucine-rich α-2-glycoprotein 1 (LRG1)-enriched TEC-derived exosomes activated macrophages through a TGFβR1-dependent process, while tumor necrosis factor-related apoptosis-inducing ligand (TRAIL)-enriched macrophage-derived exosomes induced apoptosis in injured TECs via a death receptor 5 (DR5)-dependent process, [145]. Moreover, macrophage-derived exosomes containing TRAIL induce renal tubular epithelial cell apoptosis in a TRAIL-DR5-dependent manner [145]. In another study, TEC-derived exosomes induced macrophage glycolysis by stabilizing HIF-1α in mice with DKD [150].

### 4.4. Macrophage Phenotype Influences DKD by Secreting Inflammatory Cytokines/Chemokines

**Figure 4:**
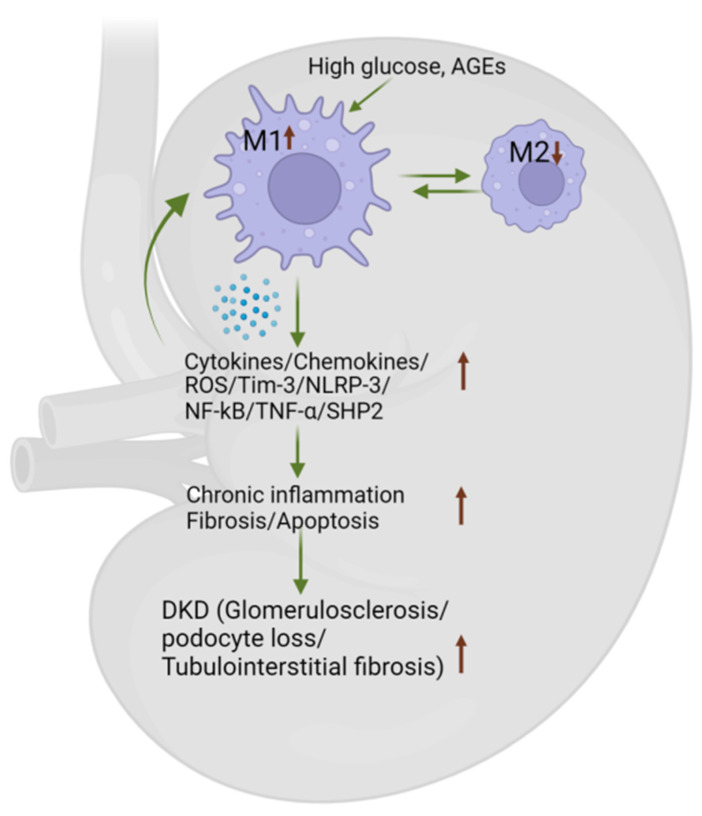
**The macrophage phenotype influences DKD by secreting inflammatory cytokines/chemokines.** Proinflammatory (M1) macrophages in the kidneys correlate with declining renal function. Inflammatory cytokines, such as TNF-α and SHP2, produced by M1 macrophages, contribute to renal damage. Additionally, Tim-3 has been identified as a key regulator of macrophage activation in the progression of DKD. NLRP-3 activation in M1 macrophages triggers NF-κB, which exacerbates renal injury. However, systemic NLRP-3 activation and increased ROS production further drive the M1-like phenotype, intensifying the severity of DKD. DKD—diabetic kidney disease, ROS—reactive oxygen species, Tim-3—T cell immunoglobulin domain and mucin domain-3, NLRP-3—NLR family pyrin domain containing-3, NF-kB—Nuclear factor-kappa B, TNF-α—tumor necrosis factor α, SHP2—Src homology-2 domain-containing protein tyrosine phosphatase-2.

Macrophages possess the ability to modulate DKD through changes in their phenotypes [151]. Macrophage phenotypes generally include a non-polarized M0 state, a pro-inflammatory M1 state, and a pro-healing M2 state [152]. It is widely recognized that M1 macrophages contribute to the initiation of renal inflammation [151,153,154,155]. The M1/M2 macrophage ratio changes dynamically throughout the progression of diabetic nephropathy (DN). It is highest in the early stages, while M2 macrophages predominate in the later stages (Stage III), resulting in the lowest M1/M2 ratio at this point [111,156]. In the early stages of T2D, M1 macrophages are recruited to the kidney and are considered to be pathogenic in DKD due to their production of proinflammatory cytokines [119,157,158,159,160,161,162,163]. Renal tissue biopsies from DN patients revealed M1 expression in glomeruli and the interstitium, with M2 and TREM-1 accumulation mainly in the interstitium. In the DN group, infiltrating CD68, M1, and M2 macrophages increased compared to controls, correlating with declining renal function over time [156]. Furthermore, TNF-α and Src homology 2–containing protein tyrosine phosphatase 2 (SHP2), derived from M1 macrophages, have been implicated in diabetic renal injury in mice [162,164]. Selective removal of TNF-α in macrophages markedly decreased albuminuria, elevation in plasma creatinine and blood urea nitrogen levels, histopathological alterations, and kidney macrophage recruitment in STZ-induced diabetic control mice [162]. Similarly, SHP2 activity was positively correlated with ERK/NF-κB activation in DKD patients [165]. Likewise, Tim-3 has also been identified as a regulator of macrophage activation during the progression of DKD. In both patients and diabetic mouse models, Tim-3 is highly expressed in renal macrophages and is positively correlated with renal dysfunction [109]. Mice lacking Tim-3 exhibit inhibition of STZ-induced DKD, while the adoptive transfer of Tim-3-expressing macrophages, but not Tim-3 knockout macrophages, accelerates diabetic renal injury [109] (Figure 4).

Recently, the NLRP-3 inflammasome, a complex of multiple proteins crucial in modulating the innate immune system and inflammatory signaling, has been implicated in both M1 and M2 macrophage polarization via distinct pathways [92,166,167]. Significantly elevated levels of NLRP-3 expression have been detected in kidney biopsies of patients diagnosed with both acute kidney injury and CKD [168]. Animal studies indicate that NLRP-3 activation in M1 macrophages triggers NF-κB, promoting renal injury [169,170] while inhibiting NF-κB activation in macrophages shifts their polarization toward M2, reducing renal injury [170,171]. NLRP-3 can also promote an M2 phenotype by increasing IL-4 expression and activating the IL-4/STAT6 pathway in a unilateral ureteral obstruction mice model [172]. It has been shown that systemic NLRP-3 inflammasome activation is critical in DKD progression [173]. NLRP-3 deficiency reduces acute kidney injury from lipopolysaccharide by suppressing renal inflammation and ferroptosis in mice [173]. Using mouse models of T1 and T2D, it was demonstrated that inhibiting NLRP-3 inflammasome activation reduces renal inflammation and fibrosis, partly by suppressing oxidative stress in DKD [174]. Moreover, the elevated production of ROS is linked to the promotion of an M1-like macrophage phenotype that contributes to advancing diabetic complications, such as DKD [175].

## 5. Macrophages as Potential Therapeutic Targets in DKD

**Figure 5:**
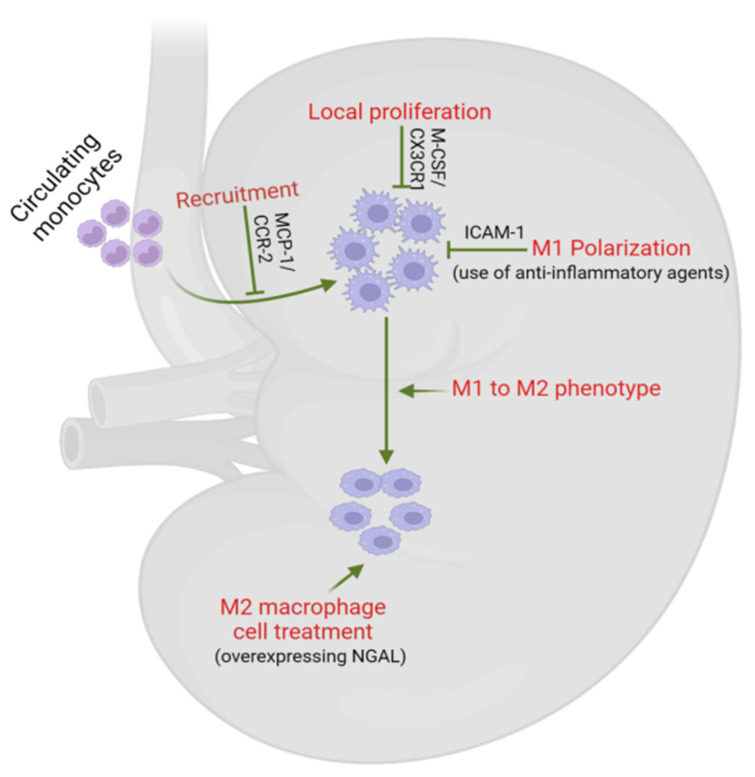
**Macrophages as potential therapeutic targets in DKD.** Macrophages can be targeted in DKD by reducing their recruitment and local proliferation, inhibiting M1 polarization/promoting M2 phenotype polarization, and using ex vivo macrophage transfusions. Strategies include inhibiting the MCP-1/CCR-2 axis and regulating local macrophage proliferation linked to renal M-CSF expression. Infusing macrophages overexpressing NGAL reduces kidney fibrosis and inflammation in chronic nephropathies, highlighting the potential of genetically modified macrophages for treating DKD. DKD—diabetic kidney disease, MCP-1/CCR-2—Monocyte chemoattractant protein-1/C-C motif chemokine receptor 2, M-CSF—macrophage colony-stimulating factor, CX3CR1—CX3C motif chemokine receptor 1, ICAM-1—Intercellular Adhesion Molecule 1, NGAL—Neutrophil gelatinase-associated lipocalin.

Focusing on inflammation and fibrosis as therapeutic targets in DKD has become a prominent area of interest, recently explored in a comprehensive review [176]. We delve deeper into macrophage-mediated inflammation in DKD. Macrophages have emerged as promising therapeutic targets in the context of DKD. They can be targeted in DKD in several ways, such as decreasing macrophage recruitment, polarizing macrophages toward the M2 phenotype, and utilizing ex vivo macrophage transfusions [177] (Figure 5). Strategies involve limiting macrophage recruitment and activation, often by inhibiting the MCP-1/CCR-2 axis. In mice deficient in CCR-2 and MCP-1, reduced urinary albumin excretion has been observed, which was linked with diminished macrophage infiltration in both T1 and T2D [158,161,178,179]. Pharmacological inhibition of MCP-1/CCR-2 resulted in a decrease in the expression of ECM components, such as fibronectin, type IV collagen, and TGF-β, normalization of IL-12 and IL-10 production, and a reduction in the number of macrophages infiltrating renal tissue in DKD in mice [179,180,181,182]. Various compounds and medications have shown efficacy in reducing macrophage infiltration and associated inflammation, ultimately preventing DKD progression in rat models of T2D [183,184]. Additionally, downregulating adhesion molecules, like ICAM-1, and utilizing specific anti-inflammatory agents further attenuate inflammatory macrophage activation, offering potential implications for DKD management [185,186,187]. Targeting key factors, such as macrophage migration inhibitory factors, holds promise in mitigating various aspects of DKD [188,189].

In patients with DKD, macrophages accumulate within the glomeruli and interstitium, and the severity of this interstitial accumulation is proportionally correlated with the rate of subsequent decline in renal function [190]. This accumulation occurs due to increased intrarenal proliferation. In a unilateral ureteral obstruction (UUO) mouse model, Engel et al. [191] demonstrated that tissue macrophage numbers are regulated through local proliferation, with CX3CR1 identified as a key regulator. Additionally, renal biopsy studies from patients have shown that local macrophage proliferation correlates with increased renal M-CSF expression in human glomerulonephritis [192]. Therefore, regulating renal macrophage proliferation may offer a potential treatment avenue for DKD.

Enhancing M2 macrophage polarization presents a potential avenue for improving DKD. In the kidneys of DN patients, TREM-1, p-STAT-1, and M1 markers (iNOS and TNF-α) are increased and positively correlated with the disease [193]. Similarly, in a rat model of DN, these markers with glomerular changes are elevated but decrease with active vitamin D3 and calcitriol treatment [193]. Furthermore, in vitro experiments suggest that active vitamin D inhibits macrophage transition to the M1 phenotype via the STAT-1/TREM-1 pathway [193]. Another study suggests that mesenchymal stem cells (MSCs) induce macrophages to transition into the M2 phenotype by enhancing transcription factor EB-mediated autophagy, which inhibits DN in mice [111,193]. Umbilical cord MSCs were found to shift macrophage polarization from pro-inflammatory M1 to anti-inflammatory M2 phenotypes through miR-146a-5p-mediated inhibition of the TRAF6/STAT1 signaling pathway [194]. Furthermore, microRNAs, like miR-223, serve as crucial regulators of macrophage plasticity, offering potential targets for mitigating DKD progression [195,196].

The utilization of M2 macrophages as a therapeutic intervention for chronic inflammatory renal conditions also shows promise. In a mouse model of DN, it was shown that M2 macrophages, produced ex vivo through IL-4 and IL-13 cytokine stimulation, influenced DN by alleviating renal structural damage. This was evidenced by diminished glomerular hypertrophy, tubular atrophy, interstitial inflammation, and fibrosis. Furthermore, a reduction in immune cell infiltration was observed in both the renal cortex and pancreas [197]. However, while splenic M2 macrophages showed protective effects, bone marrow-derived M2 macrophages did not improve renal function, suggesting differences in their response to inflammation [198]. Challenges in macrophage plasticity were also encountered. However, Roser et al. modified macrophages for stability using an adenoviral vector containing neutrophil gelatinase-associated lipocalin-2 (NGAL) and subsequently administered it to UUO mice. The macrophages were labeled and monitored in vivo. The findings showed that some infused macrophages successfully reached the obstructed kidney. Furthermore, the infusion of macrophages overexpressing NGAL was associated with decreased interstitial fibrosis and inflammation in the kidney [199]. These findings suggest the potential of genetically modified macrophages in combating chronic nephropathies.

## 6. Conclusions

It is increasingly recognized that macrophages, a crucial component of the immune system, play dual roles in the onset and progression of T2D and DKD. Unhealthy lifestyles characterized by sedentary behavior, poor dietary habits, and obesity trigger a chronic low-grade inflammation known to precede the development of T2D. Within this inflammatory milieu, macrophages play a central role. They infiltrate adipose tissue, releasing pro-inflammatory cytokines and perpetuating insulin resistance, a hallmark of T2D. As T2D ensues, macrophages continue to contribute to disease progression, infiltrating various tissues, including the kidneys. In DKD, macrophages exacerbate kidney damage through the release of inflammatory mediators, promotion of fibrosis, and disruption of normal renal function. Thus, the journey of macrophages from the initial inflammatory milieu induced by unhealthy lifestyles to their contribution to the development and progression of DKD underscores the intricate interplay between immune dysregulation and metabolic dysfunction in the pathogenesis of T2D and DKD. Efforts to target macrophage-driven inflammation may hold promise in mitigating the burden of DKD.

## 7. Future Directions

The future direction of research on macrophage-mediated inflammation in DKD holds great potential for advancing our understanding and for developing effective therapeutic interventions. One key area of focus includes exploring the plasticity of macrophage phenotypes in response to different microenvironmental cues within the diabetic kidney. Another area of focus involves the mechanism of macrophage recruitment and proliferation in diabetic renal tissue. Furthermore, understanding how macrophages contribute to renal inflammation and fibrosis in DKD could reveal novel targets for pharmacological modulation. Future research may aim to elucidate the signaling pathways and molecular mechanisms that regulate macrophage activation and polarization in DKD. Targeting these pathways could offer new therapeutic strategies to modulate macrophage function and attenuate renal inflammation. In addition, exploring the interplay between macrophages and other immune cells, such as T cells and dendritic cells, within the diabetic kidney microenvironment may uncover complex immune regulatory networks that drive disease progression. Advances in single-cell technologies, including single-cell RNA sequencing and spatial transcriptomics, will likely facilitate a more comprehensive characterization of macrophage heterogeneity and its spatial distribution within diabetic kidneys. This deeper phenotypic and spatial understanding of macrophages in DKD could inform the development of precision medicine approaches tailored to target specific macrophage populations or pathways implicated in disease pathogenesis. Ultimately, the future direction of macrophage-mediated inflammation research in DKD holds immense potential to translate scientific insights into innovative therapies that improve outcomes for patients with this debilitating complication of DKD.

## Data Availability

Not applicable.
